# Amino Acid Transporters and Glutamine Metabolism in Breast Cancer

**DOI:** 10.3390/ijms19030907

**Published:** 2018-03-19

**Authors:** Yoon Jin Cha, Eun-Sol Kim, Ja Seung Koo

**Affiliations:** Department of Pathology, Yonsei University College of Medicine, Seoul, 03722, Korea; yooncha@yuhs.ac (Y.J.C.); kesol13@yuhs.ac (E.-S.K.)

**Keywords:** amino acid transporters, human pathology, breast cancer, transport mechanism

## Abstract

Amino acid transporters are membrane transport proteins, most of which are members of the solute carrier families. Amino acids are essential for the survival of all types of cells, including tumor cells, which have an increased demand for nutrients to facilitate proliferation and cancer progression. Breast cancer is the most common malignancy in women worldwide and is still associated with high mortality rates, despite improved treatment strategies. Recent studies have demonstrated that the amino acid metabolic pathway is altered in breast cancer and that amino acid transporters affect tumor growth and progression. In breast cancer, glutamine is one of the key nutrients, and glutamine metabolism is closely related to the amino acid transporters. In this review, we focus on amino acid transporters and their roles in breast cancer. We also highlight the different subsets of upregulated amino acid transporters in breast cancer and discuss their potential applications as treatment targets, cancer imaging tracers, and drug delivery components. Glutamine metabolism as well as its regulation and therapeutic implication in breast cancer are also discussed.

## 1. Introduction

Breast cancer is the most common malignancy and second leading cause of death in women worldwide [1]. With improvements in the accuracy of diagnosis and the development of novel therapeutic agents, mortality from breast cancer has decreased. However, there are still no effective treatment strategies other than surgery for certain subsets of breast cancer, particularity triple-negative breast cancer (TNBC) [2]. Thus, researchers have aimed to identify novel therapeutic targets for breast cancer, and amino acid transporters have emerged as promising candidates.

Amino acids are essential nutrients in all living cells and are important for the proliferation and maintenance of tumor cells. Tumor cells have greater amino acid demand than normal cells since they proliferate more rapidly. In humans, some essential amino acids, including Thr, Met, Phe, Trp, Val, Ile, Leu, and Lys, must be supplied exogenously because they cannot be synthesized de novo. Tumor cells have increased demand for amino acids; thus, additional supply is needed to meet this demand, even for nonessential amino acids that are generated within the body [3]. Amino acids are the main molecules required for protein synthesis and have various other functions. Serine is a source of materials for nucleotide synthesis and DNA methylation; leucine, glutamine, and arginine are signaling factors that activate the mammalian target of rapamycin (mTOR) pathway; and glutamine, glycine, and aspartate are required for nucleotide synthesis [4,5]. Hence, the proliferation and maintenance of tumor cells are dependent on the amino acid supply to the intracellular space, which is regulated by amino acid transporters. Additionally, recent studies have demonstrated that some amino acid metabolic pathways, such as glutamine, serine, glycine, and proline, are altered in breast cancer, suggesting that amino acid transport may also be important for breast cancer proliferation and progression [6,7,8,9,10].

Cancer cells have different metabolic characteristics from those of normal cells. “Metabolic reprogramming” is a hallmark of cancer cells, and one of the most well-known alternatives is the “Warburg effect”, which is a persistently activated aerobic glycolysis in cancer cells [11]. Glucose and glutamine are the most sufficient plasma nutrients and sources of carbon metabolism. Along with the “Warburg effect”, increasing metabolism and consumption of glutamine have been reported in cancer cells, which provide carbon source for building blocks and tricarboxylic acid (TCA) cycle intermediates [12,13]. Glutamine metabolism is one of metabolic reprogramming and closely related to amino acid transporters. Glutamine is a non-essential amino acid for normal cells. However, highly proliferative cancer cells use glutamine as an essential substrate for energy source, as well as for generation of nucleotides, lipids, and proteins. In breast cancer, SLC1A5 and SLC6A14 are upregulated amino acid transporters that carry glutamines [7,14,15], and therefore glutamine metabolism may largely affect tumor biology.

In this review, we discuss the roles of amino acid transporters and glutamine metabolism in breast cancer and emphasize the importance of amino acid transporter as therapeutic targets.

## 2. Amino Acid Transporters

Amino acid transporters are membrane-bound solute carrier (SLC) transporters; approximately 430 SLCs have been reported based on analysis of the human genome and have been categorized using several classification systems [16]. Based on the human genome organization (HUGO) Gene Nomenclature Committee classification, SLCs are classified into the same family when they exhibit more than 20% sequence homology, and 52 families have been identified to date [17]. By Pfam analysis, SLCs are organized into four groups: the major facilitators superfamily (16 SLC families), the amino acid/polyamine/organocation (APC) superfamily (11 SLC families), the cation:proton antiporter/anion transporter superfamily (two SLC families), and the drug/metabolite transporter superfamily (two SLC families) [18]. Pfam clans described 15 SLC families based on the phylogenetic phenotype: α (13 SLC families), β (five SLC families), δ (two SLC families), and γ (two SLC families) groups [19,20].

Among the many SCLs identified to date, SLC1, SLC3, SLC6, SLC7, SLC15, SLC17, SLC18, SLC25, SLC32, SLC36, and SLC38 have been shown to act as amino acid transporters [21]. An ad hoc classification is used to categorize amino acid transporters based on their transporter functions in various systems, as follows: A, N, ASC, L, xc^−^, and y^+^; these acronyms indicate the substrate specificity of each transporter. Capital letters and lowercase letters indicate Na^+^-dependent transporters (except for system L, system T, and proton amino acid transporters) and Na^+^-independent transporters, respectively [22]. Amino acid transporters classified by protein sequence similarity share substrate class affinity and/or driving force [23]. Typical examples include the APC superfamily, amino acid/auxin permease family, dicarboxylate/amino acid:cation (Na^+^ or H^+^) symporter family, branched chain amino acid:cation symporter family, hydroxy/aromatic amino acid permease family, branched chain amino acid exporter family, and basic amino acid antiporter family.

## 3. Amino Acid Transporters in Breast Cancer

**Figure 1:**
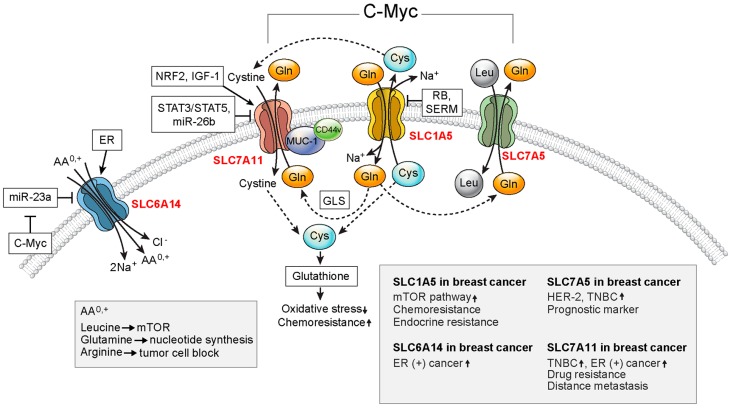
Important amino acid transporters in breast cancer. SLC1A5, SLC6A14, SLC7A5, and SLC7A11 are upregulated in breast cancer. SLC1A5, SLC7A5, and SLC7A11 exhibit functional coupling and enhance the proliferation of cancer cells. SLC7A11-mediated intracellular cysteine is used for glutathione synthesis, which results in reduced oxidative stress. c-Myc acts as a positive regulator for SLC1A5, SLC6A14, SLC7A5, and SLC7A11. STAT5: signal transducer and activator of transcription 5; ER: estrogen receptor; GLS: glutaminase; SERM: selective estrogen receptor modulator; mTOR: mammalian target of rapamycin; HER-2: human epidermal growth factor receptor 2; TNBC: triple-negative breast cancer.

Amino acid transporters are involved in nutrient supply, recycling of neurotransmitters, cell signaling pathways, and cell homeostasis [22]. Most amino acid transporters show tissue-specific and developmental stage-specific expression in normal cells [24]. Moreover, tumor cells express high levels of specific amino acid transporters according to the specific tumor type [3]. The amino acid transporters expressed in breast cancer are discussed below (Figure 1).

### 3.1. SLC1A5

SLC1A5, also known as alanine-serine-cysteine transporter 2 (ASCT2), is a Na^+^-coupled transporter for alanine, serine, cysteine, and glutamine. SLC1A5 has obligatory transport activity; one Na^+^-coupled amino acid substrate is imported into the cell, and another Na^+^-coupled amino acid substrate is exported from the cell with a 1:1 stoichiometry [25]. SLC1A5 shows higher affinity for glutamate at acidic pH [26]. Since intratumoral environments are acidic, low pH of the extracellular areas would enhance the metabolic reprogramming of tumor cells. SLC1A5 has been shown to interact with oncogenes and/or tumor-suppressor genes to mediate tumor progression. For example, SLC1A5 is a target of the oncogene c-Myc [27,28], and its expression is reduced by the tumor suppressor retinoblastoma protein [29]. Thus, SLC1A5 expression is increased in tumors with activated oncogenes and inactivated tumor suppressor genes. Functional coupling of SLC1A5 and SLC7A5 was observed in HeLa cells by transport and efflux of extracellular glutamine as a substrate for leucine uptake and mTOR activation, which are important for tumor cell growth [30]. However, a study using different type of cells (colon adenocarcinoma cell line (LS174T) and lung adenocarcinoma cell line (A549)) revealed independent SLC7A5 activity regardless of SLC1A5 status [31]. Also, Broer et al. showed that functional coupling between SLC1A5 and SLC7A5 is not obligatory; even SLC1A5 was depleted, while net glutamine uptake of HeLa cells was sustained by upregulation of SLC38A1 or SLC38A2 [32]. After cellular import of glutamine by SLC1A5, glutaminase catalyzes glutamine into glutamate, and glutamate is released from the cell by SLC7A11, which is coupled with the cellular import of cysteine [30]. Intracellular cystine is reduced to cysteine, which is used for SLC1A5-mediated glutamine transport [3]. 

In breast cancer, SLC1A5 expression varies based on the molecular subtype. In human breast cancer tissues, high SLC1A5 expression was observed in human epidermal growth factor receptor 2 (HER-2) type breast cancer by immunohistochemistry [7]. Triple-negative basal-like breast cancer cells had significantly higher SLC1A5-mediated glutamine uptake than luminal type cells [14]. In breast cancer, intracellular glutamine is important for activation of the mTOR pathway via glutaminolysis and induces cancer cell proliferation. Tamoxifen and raloxifene, two selective estrogen receptor (ER) modulators, suppress SLC1A5 expression and thereby inhibit SLC1A5-mediated glutamine uptake and proliferation of ER-negative breast cancer cells [33]. With regard to treatment resistance in breast cancer, SLC1A5 is involved in the treatment response to paclitaxel, a chemotherapy drug. Paclitaxel-induced ER stress activates the ubiquitin ligase RNF5, resulting in ubiquitination and degradation of SLC1A5 and thereby reducing glutamine uptake in breast cancer cells [34]. Diminished intracellular glutamine uptake suppresses the expression of TCA cycle intermediates, blocks mTOR signaling and tumor cell proliferation, and promotes autophagy and cell death [30,34]. Conversely, SLC1A5 upregulation in breast cancer cells decreases paclitaxel responsiveness [34]. Furthermore, SLC1A5 is associated with endocrine resistance in breast cancer cells. In aromatase inhibitor-resistant breast cancer, overexpression of the oncogene MYC upregulates SLC1A5 expression via crosstalk between ER and HER-2 [35].

As described above, SLC1A5 is associated with breast cancer through various pathways and is an independent prognostic factor for breast cancer, as demonstrated by proteomic profiling analysis [36].

### 3.2. SLC6A14

SLC6A14 (also known as ATB^0,+^) has unique features that differentiate it from other amino acid transporters. SLC6A14-mediated transport involves transmembrane gradients of Na^+^ and Cl^−^ and is coupled to membrane potential, which results in highly concentrated transport [37]. SLC6A14 (has broad affinity for all neutral and cationic amino acid substrates. SLC6A14 induces unidirectional influx of substrates, not obligatory exchange.

SLC6A14 is highly expressed in ER-positive breast cancer, as demonstrated using both primary breast cancer tissues and breast cancer cell lines [15]. Because leucine (an activator of mTOR), glutamine (an essential amino acid for nucleotide biosynthesis and substrate for glutaminolysis), and arginine (an essential amino acid for tumor cells) are critical for the proliferation of ER-positive breast cancer cells, tumor cells may upregulate SLC6A14 to meet the increased demand for these amino acids [15]. Furthermore, SLC6A14 enhances the growth of ER-positive breast cancer in spontaneous mouse models of breast cancer; this mechanism may be associated with mTOR signaling [38]. SLC6A14 is a target of the ER [15], and this may explain the relationships between SLC6A14 and ER status in breast cancer. c-Myc also modulates the expression of SLC6A14 via miR-23a inhibition [27] because SLC6A14 is a target of miR-23a [39].

### 3.3. SLC7A5

SLC7A5, also known as L-amino acid transporter 1 (LAT1), is a systemic L amino acid transporter that carries branched-chain amino acids (Val, Ile, and Leu) and bulky amino acids (Phe, Tyr, Trp, Gln, Asn, and Met) [40]. SLC3A2 (also known as 4F2hc)/SLC7A5 is a Na^+^-independent, pH-independent obligatory exchanger and is expressed in various types of tumors [41,42]. 

SLC7A5 is highly expressed in the breast cancer cell lines MCF-7 and MDA-MB-231 [43,44], with higher upregulation in MCF-7 cells than in MDA-MB-231 cells [44]. Immunohistochemical analysis of human breast cancer tissues has shown that HER-2 and TNBC types show higher expression of SLC7A5 than luminal A and B types [45]. SLC7A5 expression is associated with increased tumor size, high nuclear grade, high Ki-67 labeling index, ER negativity, and progesterone receptor (PR) negativity in breast cancer [45]. One meta-analysis investigating gene expression-based biomarkers in breast cancer revealed that SLC7A5 is a prognostic factor for breast cancer [46]. Moreover, SLC7A5 is included in one prognosis prediction test for breast cancer, called the Mammostrat test, which is an immunohistochemical multigene assay that analyzes five genes (*p53*, *HTF9C*, *CEACAM5*, *NDRG1*, and *SLC7A5*) [47]. The Mammostrat test helps to estimate the risk of early relapse in postmenopausal patients with ER-positive breast cancer treated with tamoxifen or exemestane [47] and predicts poor outcomes in patients with ER-positive breast cancer [48]. Moreover, the Mammostrat test can predict recurrence risk in patients with ER-positive, node-negative breast cancer who have undergone tamoxifen treatment [49] and reflects chemosensitivity [50]. In locally advanced breast cancer, SLC7A5 and carcinoembryonic antigen-related adhesion molecules (CEACAM5 and CEACAM6) predict poor response of neoadjuvant chemotherapy [51].

### 3.4. SLC7A11

SLC7A11 (also known as catalytic subunit of transport system x_c_^−^ or xCT), together with SLC3A2, functions as a Na^+^-independent obligatory exchanger that imports extracellular cystine (Cys-S-S-Cys) and induces the efflux of intracellular glutamate [40]. Intracellular cysteine supplied by SLC7A11 is a rate-limiting amino acid in glutathione synthesis, which is critical for antioxidant status [3]. Glutamate released into the extracellular environment by SLC7A11 plays an important role in various biological functions [52]. Moreover, high levels of SLC7A11 expression have been observed in several types of cancer. In cancer cells, increased cysteine influx and glutathione synthesis reduce oxidative damage and suppress apoptosis [53]. Extracellular glutamine also affects cancer growth. Glutamate acts through metabotropic glutamate receptors and ionotropic glutamate receptors on cancer cells and activates oncogenic signaling [54,55].

In breast cancer, SLC7A11 is important for the proliferation of TNBC [56,57]. Most breast cancer cells can survive under glutamine restriction; however, a subgroup of TNBC exhibits features of a true glutamine auxotroph, requiring cysteine import via SLC7A11 [57]. SLC7A11 has functional interactions with CD44v and mucin 1 transmembrane C-terminal subunit (MUC1-C). MUC1-C binds directly to CD44v and enhances the stability of SLC7A11 [56]. Conversely, elevated extracellular glutamate concentration suppresses SLC7A11 in TNBC cells, which induces intracellular cysteine depletion and results in the accumulation and activation of hypoxia inducible factor 1 (HIF1) α. This phenomenon appears to be one of the mechanisms for HIF1 α expression in TNBC by glutamate efflux. The expression of SLC7A11 is upregulated in ER-positive breast cancer cells, such as MCF-7 cells, and in TNBC, which allows adaptation under oxidative stress conditions by increasing glutathione synthesis via increased cysteine influx [58,59]. Nuclear factor erythroid 2-related factor 2 [58] and insulin-like growth factor-1 [59] are positive regulators that upregulate SLC7A11 expression. In contrast, signal transducer and activator of transcription (STAT) 3/STAT5 [60,61] and miR-26b [62] are negative regulators that inhibit SLC7A11 expression in breast cancer. 

SLC7A11 is associated with treatment resistance in breast cancer. Adriamycin suppresses SLC7A11 activity and causes overexpression of reactive oxygen species (ROS)-induced P-glycoprotein, resulting in drug resistance [63]. SLC7A11 is also responsible for distant metastasis of breast cancer. In a mouse model with brain metastasis from breast cancer, SLC7A11 was found to be upregulated [64]. In breast cancer cells, SLC7A11 was shown to be upregulated in tumorspheres with cancer stem cell features and to affect pulmonary metastasis [65].

### 3.5. Other SLCs

SLC3A1 (also known as rBAT) is a Na^+^-independent transporter of cystine and neutral and dibasic amino acids, which has been reported to be associated with breast cancer tumorigenesis [66]. Upregulation of SLC3A1 increases cysteine uptake and synthesis of reductive glutathione, which reduces ROS, activates protein phosphatase 2, and results in activation of the AKT signaling pathway [66]. SLC7A7 (also known as y + LAT1) uptakes dibasic amino acids in a Na^+^-independent manner and involves Na^+^-dependent uptake of some neutral amino acids. It is identified by an expression change of up to 6.5-fold after PR transfection in the TNBC cell line of MDA-MB-231 cells, suggesting that SLC7A7 is a target of PR [67]. Moreover, overexpression of SLC7A7 has been reported in HER-2 positive breast cancer cells lines [68]. SLC7A8, which is a Na^+^-independent, large neutral amino acid transporter 2 (LAT2), has been reported as a predictive biomarker of good response to endocrine therapy in ER-positive breast cancer [69,70]. SLC38A1, also known as sodium-coupled neutral amino acid transporter 1 (SNAT1), is upregulated in both breast cancer cell lines (two originated from human (MCF-7, MDA-MB-231) and one from mouse (4T1)) and human breast cancer tissues [57]. Overexpression of SLC38A1 is related to tumor size, nodal metastasis, advanced tumor stage, Ki-67 expression, and ER status [71]. Because SLC38A1 overexpression is correlated with increased phosphorylation of AKT, crosstalk between SLC38A1 and AKT signaling may affect breast cancer progression [71].

## 4. Clinical Applications of Amino Acid Transporters in Breast Cancer

### 4.1. Amino Acid Transporters as Therapeutic Targets

Because various amino acid transporters are overexpressed in breast cancer, they may be potential therapeutic targets. Theoretically, inhibition of amino acid transporters in cancer cells induces nutrient depletion and leads to growth inhibition and death of cancer cells [72,73]. Although cancer cells exhibit higher expression of amino acid transporters than normal cells, normal cells are also largely dependent on amino acid transporters for nutrient supply. Therefore, it is important to estimate the effects of amino acid transporter inhibitors in normal cells, and knockout mouse models have proven suitable for such evaluations. In previous studies, mice with deficiencies in SLC1A5, SLC6A14, and SLC7A11, which have been reported to be upregulated in breast cancer, were found to be viable and fertile and to have no abnormal phenotypes [38,74,75]. In contrast, *Slc7a5* knockout was shown to be embryonically lethal [76]. This result may be related to the role of SLC7A5 in transmitting amino acids through the blood-brain barrier to facilitate brain development; alternatively, SLC7A5 is also highly expressed in the placenta and plays an important role in fetal development [42]. However, SLC7A5-selective inhibitor JPH203 has been shown to induce growth inhibition of tumor cells in different human cell lines and mouse models [77,78]. These in vivo studies showed that JPH203 was non-toxic to normal cells, thereby suggesting that SLC7A5 was a potential therapeutic target [77,78]. Elective inhibitors of amino acid transporters that are upregulated in breast cancer are shown in Table 1.

As the active form of vitamin D, 1,25-dihydroxyvitamin D (1,25(OH)2D) downregulates SLC1A5 and reduces glutamine uptake and utilization in Harvey-ras oncogene-transformed MCF10A human breast epithelial cells, potentially contributing to breast cancer prevention. This mechanism is possible because the *SLC1A5* gene promoter harbors a vitamin D response element [79]. Treatment of ER-positive breast cancer cells with α-methyl-dl-tryptophan (α-MT), a selective blocker of SLC6A14, leads to amino acid deprivation, inhibition of the mTOR pathway, activation of autophagy, and induction of cancer cell apoptosis. The effects of α-MT persist in mouse xenograft models and in vitro cell lines [15]. The nonmetabolizable amino acid 2-amino-2-norbornane-carboxylic acid, which is an inhibitor of system L amino acid transporters, inhibits WST-1 metabolism in breast cancer cell lines (MCF-7, ZR-75-1, and MDA-MB-231) and suppresses cell growth in a concentration-dependent manner [80]. Sulfasalazine, an SLC7A11 inhibitor, increases intracellular glutamate levels in TNBC cell lines and inhibits MUC1 expression. Erastin, another SLC7A11 inhibitor, induces iron-dependent cell death, known as ferroptosis, in TNBC cells exhibiting MUC1-C suppression [56]. Sulfasalazine also dramatically reduces culture size in TNBC cell lines [57] and enhances the chemoresponsiveness of breast cancer cells to doxorubicin [81]. Sulfasalazine is an SLC3A1 inhibitor that suppresses the breast cancer growth response to antioxidant *N*-acetylcysteine [66].

### 4.2. Cancer Imaging

Overexpression of amino acid transporters in breast cancer cells can be utilized in cancer imaging by positron emission tomography-computed tomography (PET-CT). Current PET-CT technologies use ^18^F-deoxyglucose as a tracer for cancer imaging, based on the tumor-specific upregulation of glucose transporter-1 (GLUT-1; also known as SLC2A1) on tumor cells [82]. Hence, breast cancer-specific amino acid transporter substrates may have applications as tracers for PET imaging of breast cancer. Common amino acid tracers used for breast cancer in preclinical studies are shown in Table 1.

2-Amino-5-(4-[^18^F]fluorophenyl)pent-4-ynoic acid ([^18^F]FPhPA) is a synthetic amino acid that targets SLC1A5 and SLC7A5. High radiotracer uptake of [^18^F]FPhPA was found in the murine breast cancer cell line EMT6 by PET imaging [83]. *Trans*-1-amino-3-^18^F-fluorocyclobutanecarboxylic acid (*anti*-^18^F-FACBC, also known as ^18^F-fluciclovine) is a synthetic l-leucine analogue. Previous studies using prostate cancer cell lines elucidated that SLC1A5 is a major transporter of ^18^F-fluciclovine [84,85], and that SLC7A5 is an important transporter in an acidic environment [86]. PET imaging using ^18^F-fluciclovine showed significantly higher SUV_max_ in breast cancer compared with that in benign breast lesions. In breast cancer, ^18^F-fluciclovine uptake was correlated with triple-negative receptor status and nuclear grade 3 [87]. ^18^F-fluciclovine has also been used to detect unsuspected extra-axillary nodal metastases of breast cancer and had a high SUV_max_ in invasive lobular carcinoma [88]. *O*-(2-^18^F-fluoroethyl)-l-tyrosine (^18^F-FET) is a synthetic amino acid transported by SLC7A5. Animal experiments using rats and mice showed that ^18^F-FET PET could distinguish between inflammation and malignant tumor [89,90]. In human cancers, ^18^F-FET PET was positive in 75% of breast cancer patients. Meanwhile, activation and differentiation of T-cells were coupled with SLC1A5- and SLC7A5-dependent glutamine uptake [75,91]. Therefore, when it comes to breast cancer subsets with high levels of tumor-infiltrating lymphocytes, most of them are T-cells that could have more intense uptake in ^18^F-FET PET. Conversely, SLC1A5 and/or SLC7A5-expressing T-cells in inflammatory disease or autoimmune disease would lead to false-positive results in ^18^F-FET PET images. 

(4*S*)-4-(3-[^18^F]fluoropropyl)-l-glutamate (BAY 94-9392, also known as [^18^F]FSPG) is a synthetic amino acid analog of SLC7A11. [^18^F]FSPG PET identified approximately 40% of [^18^F]FDG lesions in breast cancer, with a lower SUVmax than that of [18F]FDG [92]. ^18^F-5-fluoroaminosuberic acid, a synthetic amino acid substrate of SLC7A11, exhibited tumor uptake in three breast cancer cells lines (MDA-MB-231, MCF-7, and ZR-75-1), with the highest uptake observed in MDA-MB-231, a TNBC cell line [93].

Amino acid transporters can be also used for single-photon emission computed tomography (SPECT) imaging of tumors. 3-[^123^I] Iodo-α-methyl-l-tyrosine (IMT) is an artificial amino acid that is transported via SLC7A5 [94], as well as a suitable metabolic tracer for SPECT in extracranial tumors including breast cancer [95]. With IMT SPECT, primary and metastatic breast cancer and regression of tumor after radiotherapy were all detectable and concordant with the clinical assessment results [95]. Scintimammography using [99m]Tc-labeled diethylenetriaminepentaaceticacid (DTPA-bis)-methionine showed 96% sensitivity and 96% positive predictive value for breast cancer detection [96] and could therefore be an alternative to conventional SPECT using nonspecific mitochondrial uptake.

### 4.3. Drug Delivery

Amino acid transporters can deliver drugs specifically to cancer cells. Modified chemotherapeutic agents can be delivered as substrates via specific amino acid transporters. One such modification method involves the use of nanoparticles. An in vivo study showed that paclitaxel-loaded SLC7A5-targeting poly(lactic-*co*-glycolic acid) nanoparticles exhibited cytotoxicity, cellular uptake, and in vivo antitumor effects in breast cancer [97]. Multibranched gold nanoparticles (AuNPs) conjugated to catechol-containing SLC7A5 ligands, l-dopa, and d-dopa were found to be highly accumulated in various breast cancer cell lines (MCF-7, MDA-MB-231, MDA-MB-468, and MDA-MB-453). Moreover, Ag^+^ can be used during the development of AuNPs to achieve strong near-infrared absorbance, thereby inducing selective photothermal ablation in TNBC cells (MDA-MB-231 cells) and enhancing responses to the anticancer drugs cisplatin and docetaxel [98].

## 5. Glutamine Metabolism in Breast Cancer

Metabolic pathways in cancer cells are widely heterogeneous and complex, affecting each other via numerous interactions and networks of subcellular structures. In this section, we discuss glutamine metabolism, which is a distinct metabolism related to amino acid transporters. 

### 5.1. Role of Glutamine and Glutamine Metabolism in Breast Cancer Cells

Glutamine supports the proliferation and progression of cancer cells. First, glutamine is a substrate for nitrogen, which is required for the synthesis of nucleotides and non-essential amino acids [99]. Second, glutamine provides a carbon source for the TCA cycle for citrate and fatty acid synthesis via α-ketoglutarate (α-KG) production. [13,100]. Third, glutamine is involved in glutathione synthesis and maintains redox balance [101]. Last, glutamine activates the mTOR pathway by enhancing amino acid influx, including leucine [102]. In addition, mTORC1 also could be activated by glutaminolysis, particularly gruanosine triphosphate (GTP) loading of Rag, which is a downstream molecule of glutaminolysis [103]. Intracellular glutamine is converted into glutamate, a precursor of α-KG, by glutaminase (GLS1). Glutamate is a precursor for most non-essential amino acids, like Asp, Ala, Arg, and Pro [104]. Overexpression of glutaminase (GLS1) in cancer cells and a subsequent increase of amino acids production have been reported in ER-negative breast cancer, with apparent association to poor prognosis [105]. Some subsets of breast cancer, especially MYC-overexpressing tumors most of which are TNBC, show glutamine addiction, which could be a potential therapeutic target [4,57,106].

### 5.2. Regulation of Glutamine Metabolism in Breast Cancer

Metabolic reprogramming of cancer cells is regulated by oncogenes and/or tumor suppressor genes, or by alteration in copy number of metabolism-related genes. [107,108]. c-Myc oncogene amplification is found in approximately 15% of breast cancers, particularly more in basal-like type breast cancers [109]. c-Myc affects the regulation of glutamine metabolism. c-Myc binds to the promoter of SLC1A5, a glutamine transporter, and induces overexpression of SLC1A5 and glutamine influx [27,28]. c-Myc inhibits miR-23a and miR-23a/b, and results in the activation of SLC6A14 [27] and the overexpression of glutaminase (GLS1) [27,28], respectively. Glutamine uptake is also controlled by Rb, a tumor suppressor gene. Wild-type Rb inhibits SLC1A5 expression through E3F-3 mediated regulation of the SLC1A5 promoter [29].

### 5.3. Alteration of Glutamine Metabolism in Breast Cancer According to Molecular Subtypes

Breast cancer is a heterogeneous disease with heterogeneity in histology, molecular profiles, clinical course, and treatment response. Although histologic classification has been generally used in breast cancer classification, several molecular classifications are now adopted as new molecular analytical techniques have been introduced. The traditionally used molecular classification divided breast cancer into luminal A, luminal B, HER-2-enriched, and basal-like types (most as TNBC) [110,111]. Along with gene expression patterns, clinical features and treatment responses differ among the intrinsic molecular subtypes [110,111]. Therefore, different metabolic characteristics are expected according to the molecular subtype. Previous studies have shown different metabolic signatures between ER-positive and ER-negative breast cancers [112,113]. 

High expressions of enzymes of glutamine metabolism have been reported in TNBC [14]. Expressions of GLS and glutamate dehydrogenase (GDH) were low in luminal A type, and high in HER-2 type [7]. HER-2 positive breast cancer and TNBC type had higher glutamate levels and lower glutamine levels compared to ER-positive breast cancer [108,114]. Therefore, HER-2 positive breast cancer and TNBC are expected to have high glutamine influx and active glutaminolysis.

### 5.4. Therapeutic Implication of Glutamine Metabolism in Breast Cancer

As glutamine metabolism is activated in HER-2 positive breast cancer and TNBC, it might be a therapeutic target. CB-839, a GLS inhibitor, has antitumor activity in TNBC cells [115]. Bis-2-(5-phenylacetamido-1,2,4-thiadiazol-2-yl)ethyl sulfide (BPTES) specifically inhibits GLS1 and enhances the therapeutic efficacy of cisplatin or etoposide in TNBC cells [116]. In addition, c-Myc suppression could have therapeutic implications. CX-3543 is a small molecule inhibitor that binds to the G-quadruplex of the c-Myc promoter. CX-3543 shows selective lethality in BRCA1/2 deficient cancer [117], with most of them being basal-like type or TNBC [118].

## 6. Conclusions

Breast cancer is the most commonly diagnosed cancer in women. A variety of treatment modalities, including endocrine therapy, monoclonal therapy, chemotherapy, and radiotherapy, are used in combination with surgery. Although the survival rate of patients with breast cancer has increased, further decreases in morbidity and mortality are still needed. In particular, effective therapeutic agents for TNBC are urgently required. Some amino acid transporters, including SLC1A5, SLC6A14, SLC7A5, and SLC7A11, may be promising targets for the treatment of breast cancer since they modulate tumor growth, metastasis, treatment response, and prognosis of breast cancer. In breast cancer, glutamine is a key nutrient for cancer cell proliferation and survival, and it is largely regulated by amino acid transporters, especially SLC1A5 and SLC7A5. In the context of potential target agents, active surveillance of these amino acid transporters with clinical studies is required after preclinical research. Moreover, amino acid transporters could be used as tracers for PET imaging of tumors and as mediators for nanoparticulate drug delivery systems. Activated glutamine metabolism in subsets of breast cancer—HER-2 positive breast cancer and TNBC—has clinical implication as a potential therapeutic target.

## Figures and Tables

**Table 1:** Portfolio of amino acid transporters overexpressed in breast cancer.

Gene	Synonym	Transport Mechanism	Substrates	Inhibitor	Imaging Tracer	Drug Delivery Candidate
SLC1A5	ASCT2	Obligatory exchange; Na^+^/AA exchanged for Na^+^/AA	Ala, Ser, Cys, Thr, Gln	1,25-dihydroxyvitamin D	[(18)F]FPhPA; (18)F-FACBC	
SLC6A14	ATB^0,+^	Unidirectional; Na^+^/Cl^−^/AA^0,+^ symport	All neutral amino acids; All cationic amino acids	α-methyl-dl-tryptophan		
SLC7A5	LAT1	Obligatory exchange; AA exchanged for AA	Large neutral amino acids	BCH (2-amino-2-norbornane-carboxylic acid) JHP203	[(18)F]FPhPA; (18)F-FACBC; [99m]Tc-labeled diethylenetriaminepentaaceticacid (DTPA-bis)-methionine	Polylactic-co-glycolic acid (PLGA) nanoparticles; AuNPs
SLC7A11	xCT	Obligatory exchange; AA exchanged for AA	Cystine, glutamate	Sulfasalazine; Erastin	[(18)F]FSPG ^18^F-5-FASu	
SLC3A1	rBAT	Obligatory exchange; AA exchanged for AA	cystine and neutral and dibasic amino acids			
SLC7A8	LAT2	Obligatory exchange; AA exchanged for AA	Large neutral amino acids			
SLC38A1	ATA1	Obligatory exchange; Na^+^/AA exchanged for Na^+^/AA	All neutral amino acids			
